# Improving preoperative risk-of-death prediction in surgery congenital heart defects using artificial intelligence model: A pilot study

**DOI:** 10.1371/journal.pone.0238199

**Published:** 2020-09-04

**Authors:** João Chang Junior, Fábio Binuesa, Luiz Fernando Caneo, Aida Luiza Ribeiro Turquetto, Elisandra Cristina Trevisan Calvo Arita, Aline Cristina Barbosa, Alfredo Manoel da Silva Fernandes, Evelinda Marramon Trindade, Fábio Biscegli Jatene, Paul-Eric Dossou, Marcelo Biscegli Jatene

**Affiliations:** 1 Department of Cardiovascular Surgery—Pediatric Cardiac Unit, Heart Institute of University of São Paulo Medical School—HCFMUSP—InCor, São Paulo, Brazil; 2 Fundação Armando Alvares Penteado–FAAP, São Paulo, Brazil; 3 Escola Superior de Engenharia e Gestão–ESEG, São Paulo, Brazil; 4 Health Technology Assessment Center of Clinics Hospital–NATS-HCFMUSP, São Paulo, Brazil; 5 São Paulo State Health Secretariat–SES-SP, São Paulo, Brazil; 6 Institut Catholique des Arts et Métiers–Icam, Paris-Sénart, France; Oregon Health & Science University, UNITED STATES

## Abstract

**Background:**

Congenital heart disease accounts for almost a third of all major congenital anomalies. Congenital heart defects have a significant impact on morbidity, mortality and health costs for children and adults. Research regarding the risk of pre-surgical mortality is scarce.

**Objectives:**

Our goal is to generate a predictive model calculator adapted to the regional reality focused on individual mortality prediction among patients with congenital heart disease undergoing cardiac surgery.

**Methods:**

Two thousand two hundred forty CHD consecutive patients’ data from InCor’s heart surgery program was used to develop and validate the preoperative risk-of-death prediction model of congenital patients undergoing heart surgery. There were six artificial intelligence models most cited in medical references used in this study: Multilayer Perceptron (MLP), Random Forest (RF), Extra Trees (ET), Stochastic Gradient Boosting (SGB), Ada Boost Classification (ABC) and Bag Decision Trees (BDT).

**Results:**

The top performing areas under the curve were achieved using Random Forest (0.902). Most influential predictors included previous admission to ICU, diagnostic group, patient's height, hypoplastic left heart syndrome, body mass, arterial oxygen saturation, and pulmonary atresia. These combined predictor variables represent 67.8% of importance for the risk of mortality in the Random Forest algorithm.

**Conclusions:**

The representativeness of “hospital death” is greater in patients up to 66 cm in height and body mass index below 13.0 for InCor’s patients. The proportion of “hospital death” declines with the increased arterial oxygen saturation index. Patients with prior hospitalization before surgery had higher “hospital death” rates than who did not required such intervention. The diagnoses groups having the higher fatal outcomes probability are aligned with the international literature. A web application is presented where researchers and providers can calculate predicted mortality based on the CgntSCORE on any web browser or smartphone.

## Introduction

Congenital heart defects (CHD) are structural problems that arise in the formation of the heart or major blood vessels, with a significant impact on morbidity, mortality and health costs in children and adults. Defects vary in severity, from tiny holes between chambers that are resolved naturally or malformations that may require multiple surgical procedures, being a major cause of perinatal and infant mortality [1].

Reported birth estimates for patients with congenital heart disease vary widely among studies worldwide. The estimate incidence of 9 per 1,000 live births is generally accepted, thus, annually, more than 1.35 million of children are expected to be born with some congenital heart disease [2,3].

CHD may require several surgical procedures carrying its implicit death risk [2]. Survival risk analysis provides support for medical decision-making, avoiding futile clinical interventions or ineffective treatments [4]. There are some risk stratification models for mortality and morbidity for children with congenital heart disease, for example RACHS-1 [5–7], Aristotles Basic Complexity (ABC) and Aristotles Comprehensive Complexity (ACC) [8]. These models were developed based on experience and consensus among experts in this field, due to the lack of adequate data at that time [9].

One of the greatest challenges in developing accurate predictors of death related to pediatric heart surgery is the wide heterogeneity range of congenital heart anomalies. Unlike adult cardiac surgery (where there is a limited number of surgical procedures and very large numbers of patients undergoing such procedures), the exact opposite applies in pediatric cardiac surgery where there are thousands of different procedures and small number of patients undergoing each type of procedures. Many cardiac surgical programs may undertake certain rare procedures only once in several years. Thus, to build a helpful risk predictive model, the experience of a large number of patients must be analyzed. Of note, the Society of Thoracic Surgeons (STS) has the world’s largest multi institutional congenital cardiac database registering data from the majority of United States and Canada pediatric cardiac centers [10]. The STS has published numerous comprehensive articles modelling risk factor analysis in a representative population living and treated in a developed country. Recently a new global database is being established by the World Society for Pediatric and Congenital Heart Surgery (WSPCHS). It aims to include multiple institutions from multiple countries with the proposal to represent more heterogeneous population of children assisted by different health systems and facilities [11]. Indeed, the motivation for the development of the global database is exactly the problem pointed in 2014, that we used to justify the ASSIST Registry project: there is massive heterogeneity of CHD diagnosis, procedures, patient characteristics, trained human resources and facilities diverse structures where they are treated. Following the need to perform outcomes assessments, respecting the characteristics of our population and healthcare system, we hope to establish a multi institutional Brazilian database in the near future based on our ASSIST Registry.

The ASSIST Registry was established in 2014 as a multicenter São Paulo State regional database [12], as the pilot for this national project. In the past 5 years the ASSIST Registry collects data from five institutions aiming to elicit our population’s specific covariates and their individual conditions that could modify the outcomes risk model, currently based on international well established risk scales for databases stratification [10]. The predictions of these models, despite the efforts for enhancing it are not sufficiently accurate for individual’s risk assessment worldwide, either because the performance of a given risk score reflects the average of a group or because there are sociodemographic particularities that affect the model's response [13,14]. Given this scenario, individualized mortality prediction models have been proposed [15]. Some studies with AI aids have been performing better when compared to the standard severity scoring system [16,17].

The proposal for mortality risk models using artificial intelligence for patients with congenital heart disease is promising, although research on this subject is scarce. Recent studies include machine-learning algorithms to classify groups of risks of death in surgery [16]. Another study proposed an artificial neural network (ANN) model to predict the risks of congenital heart disease in pregnant women [17]. However, there was no result when we searched the published scientific literature for specific models of individual prediction of death in cardiac surgery for patients with congenital heart defects.

The proposal of this study is to evaluate six statistics models to ascertain the mortality risk, adapted to the regional reality, focused on individual mortality prediction among patients with congenital heart disease undergoing cardiac surgery. Secondly, we aimed to instrument the model-based mortality prediction with a calculator tool, the CngtSCORE calculator model, accessible through any web browser or smartphone.

## Materials and methods

### Study design

This is a retrospective post-hoc AI analysis of the prospectively built ASSIST Registry multicenter CHD 2014–2019 study. These analyses intended to elicit the highest AI accuracy model to build the individual’s death risk prediction before individual’s surgery.

Six artificial intelligence models most cited in medical references were used in this study. The Multilayer Perceptron (MLP), Random Forest (RF), Extra Trees (ET), Stochastic Gradient Boosting (SGB), Ada Boost Classification (ABC) and Bag Decision Trees (BDT) machine-learning algorithms were tested with the InCor’s dataset aiming to elicit the most adjusted outcome evaluation.

### Study population

Between January 2014 and December 2018, there were 2,240 consecutive patients with CHD referred for InCor’s surgical treatment. All data were extracted from the general ASSIST Registry dataset and stored in compliance with institutional security and privacy governance rules.

The database ASSIST Registry accumulates more than 3,000 patients reported [12].

Despite this collaborative dataset (since the data from the remaining centers was not externally audited until now), we used only the InCor’s data to keep this pilot test data more accurate.

To ensure data accuracy, the postgraduate student and the supervisors (authors) performed quality checks over time.

### Predicting variables

Eighty-three pre-operational ASSIST Registry predictive variables for the outcome of each patient were applied. Selection decisions were made based on their methodology, the evidence literature used, their applicability, and by consensus among the participant researchers (these variables and its parametrization are presented in Table 1). These variables were used as exogenous variables in the six machine-learning algorithms to create the CgntSCORE calculator. The six algorithms trained in this study were Multilayer Perceptron (MLP), Random Forest (RF), Extra Trees (ET), Stochastic Gradient Boosting (SGB), Ada Boost Classification (ABC) and Bag Decision Trees (BDT). These six different machine-learning algorithms were used to predict the risk of pre-surgical mortality and to understand the magnitude each variable affected the risk of death.

**Table 1:** Pre-operative variables.

Variable	Characteristic	Description
PRE_proc_2	Origin (patient's home)	1—Ignored
		2—Pará
		3—São Paulo—state
		4—São Paulo—capital
		5—Espírito Santo
		6—Goiás
		7—Minas Gerais
		8—Rio Grande do Norte
		9—Tocantins
		10—Paraná
		11—Bahia
		12—Roraima
		13—Brasília
		14—Mato Grosso do Sul
		15—Pernambuco
		16—Paraíba
		17—Ceará
		18—Santa Catarina
		19—Amazonas
		20—Piauí
		21—Mato Grosso
		22—Rio de Janeiro
		23—Maranhão
		24—Rio Grande do Sul
		25—Amapá
		26—Alagoas
		27—Rondônia
		28—Acre
PRE_prov_3	Provider	1 = SUS
		2 = Particular
		3 = Health insurance
		4 = Ignored
PRE_sexo_4	Sex	1—Male
		2—Female
PRE_prenat_5	Diagnosis of Congenital Heart Disease in Prenatal Care	1 = No
		2 = Yes
		3 = No information
PRE_premat_6	Prematurity	1 = No
		2 = Yes
		3 = No information
PRE_maediab_7	Son of a Diabetic Mother	1 = No
		2 = Yes
		3 = No information
PRE_cirpre_8	Previous Surgery	1 = No
		2 = Yes
PRE_intpre_9	Number of Hospitalizations	1 to 12
PRE_ncirpre_10	Number of Previous Sugeries	1 to 7
PRE_sindcrom_11	Non-Cardiac Abnormality—Chromossomal Sydrome	1 = No
		2 = Yes
PRE_down_12	Down's Sydrome	1 = No
		2 = Yes
PRE_digeorge_13	DiGerorge Syndrome	1 = No
		2 = Yes
PRE_turnner_14	Turner Syndrome	1 = No
		2 = Yes
PRE_willians_15	Williams Syndrome	1 = No
		2 = Yes
PRE_edwards_16	Edwards Syndrome	1 = No
		2 = Yes
PRE_Noonan_17	Noonan Syndrome	1 = No
		2 = Yes
PRE_outcrom_18	Other Syndromes	1 = No
		2 = Yes
PRE_anoanat_19	Non-cardiac Abnormality—Malformations	1 = No
		2 = Yes
PRE_atresiaesofag_20	Esophageal Atresia	1 = No
		2 = Yes
PRE_anusimperf_21	Imperforated Anus	1 = No
		2 = Yes
PRE_Fistraqueo_22	Tracheoseophageal Fistula	1 = No
		2 = Yes
PRE_herniadiaf_23	Diaphragmatic Hernia	1 = No
		2 = Yes
PRE_onfalocele_24	Omphalocele	1 = No
		2 = Yes
PRE_fendaPalat_25	Cleft Palate	1 = No
		2 = Yes
PRE_Outanoanat_26	Other Anomalies	1 = No
		2 = Yes
PRE_ht_preop_adm1_27	Pre-operative Hematocrit	1. 21 to 26
		2. 27 to 32
		3. 33 to 38
		4. 39 to 44
		5. 45 to 50
		6. 51 to 56
		7. 57 to 62
		8. 63 to 70
		9. No information
PRE_sato2_preop_adm1_28	Arterial Oxygen Saturations	1. 55 to 60%
		2. 61 to 64%
		3. 65 to 67%
		4. 70 to 73%
		5. 74 to 78%
		6. 79 to 82%
		7. 83 to 87%
		8. 88 to 92%
		9. 93 to 96%
		10. 97 to 100%
		11. No information
PRE_Diagnostico_categoria1a12_29	Diagnosis Category	1 = cardiomiopatia
		2 = cor triatriatum
		3 = DORV—double outlet right ventricle
		4 = electrophysiological
		5 = left heart lesions
		6 = miscellaneous, other
		7 = pulmonary venous anomalies
		8 = right heart lesions
		9 = septal defects
		10 = single ventricle
		11 = thoracic arteries and veins
		12 = transportation of the great arteries
PRE_Truncus_30	Truncus Arteriosus	1 = No
		2 = Yes
PRE_CMP_31	Cardiomypathy	1 = No
		2 = Yes
PRE_DAPVVPP_32	Partial Anomalous Drainage of the Pulmonary Veins	1 = No
		2 = Yes
PRE_DATVVPP_33	Total Anomalous Drainage of the Pulmonary Veins	1 = No
		2 = Yes
PRE_Aneurisma_Ao_34	Aortic Aneurysm	1 = No
		2 = Yes
PRE_Doenca_ValvaAO_35	Aortic Valve Disease	1 = No
		2 = Yes
PRE_Doenca_ValvaMi_36	Mitral Valve Disease	1 = No
		2 = Yes
PRE_Doenca_ValvaTri_37	Tricuspide Valve Disease	1 = No
		2 = Yes
PRE_Janela_AO_Pulm_38	Aortopulmonary Window	1 = No
		2 = Yes
PRE_PCA_39	Persistence of the Arterial Canal	1 = No
		2 = Yes
PRE_CIA_40	Arterial Communication	1 = No
		2 = Yes
PRE_DSAV_41	Atrioventricular Septal Defect	1 = No
		2 = Yes
PRE_CIV_42	Interventricular Communication	1 = No
		2 = Yes
PRE_CoAo_HipoArcoAO_43	Aortic Arch Coartation ans Hypoplasia	1 = No
		2 = Yes
PRE_Miscelania_44	Miscellaneous	1 = No
		2 = Yes
PRE_TCGA_45	Corrected Transposition of the Great Arteries	1 = No
		2 = Yes
PRE_CorTriatriatum_46	Cor Triatriatum	1 = No
		2 = Yes
PRE_Anomalia_Coronaria_47	Coronary anomaly	1 = No
		2 = Yes
PRE_DVSVD_48	Dual Right Ventricular Outflow Tract	1 = No
		2 = Yes
PRE_SHCE_49	Left Heart Hypoplasia Syndrome	1 = No
		2 = Yes
PRE_T4F_50	Tetralogy of Fallot	1 = No
		2 = Yes
PRE_RVOT_51	Expansion of the Right Ventricular Outflow Tract	1 = No
		2 = Yes
PRE_Interrupcao_ArcoAO_52	Aortic Arch Disruption	1 = No
		2 = Yes
PRE_Atresia_pulmonar_53	Pulmonary Atresia	1 = No
		2 = Yes
PRE_Doenca_ValvaPulm_54	Pulmonary Valvopathy	1 = No
		2 = Yes
PRE_TGA_55	Transposition of the Great Arteries	1 = No
		2 = Yes
PRE_Ventriculo_unico_56	Single Ventricule	1 = No
		2 = Yes
PRE_Eletrofisiologia_57	Heart Rhythm Changes	1 = No
		2 = Yes
PRE_Tunel_VE_AO_58	Tunneling Left Ventricule Aorta	1 = No
		2 = Yes
PRE_Shone_59	Shone syndrome	1 = No
		2 = Yes
PRE_Anel_Vascular_60	Vascular Ring	1 = No
		2 = Yes
PRE_grupo_diag_61	Diagnostic Group	1 = Aortic Aneurysm
		2 = Aortic Valve Disease
		3 = AP Window
		4 = ASD
		5 = AV Canal
		6 = Cardiomyopathy
		7 = Coarctation of Aorta and Aortic Archhypoplasia
		8 = Congenitally Correted TGA
		9 = Cor Triatriatum
		10 = Coronary Artery Anomalies
		11 = DORV
		12 = Electrophysiological
		13 = Hypoplastic Left Heart Syndrome
		14 = Interrupted Arch
		15 = LV to Aorta Tunnel
		16 = Miscellaneous
		17 = Mitral Valve Disease
		18 = Partial Anomalous Pulmonary Venous Connection
		19 = Patent Ductus Arteriosus
		20 = Pulmonary Atresia
		21 = Pulmonary Valve Disease
		22 = RVOT Obstruction and/or Pulmonary Stenosis
		23 = Shone's Syndrome
		24 = Single Ventricle
		25 = Tetralogy of Fallot
		26 Total Anomalous Pulmonary Venous Connection
		27 = Transposition of the Great Arteries
		28 = Tricuspid Valve Disease and Ebstein Anormaly
		29 = Truncus Arteriosus
		30 = Vascular Rings and Slings
		31 = VSD
PRE_proc_previos_2Sim_1Nao_63	Patient Undergoing Previus Procedures	1 = No
		2 = Yes
PRE_proc_previos_01_64	Number of Previous Procedures	1 to 85
PRE_Ressuc_PCR_Pre_Adm1_65	Pre-operative ressuscitated Cardiac Arrest Patient	1 = No
		2 = Yes
PRE_Arritmia_pre_Adm1_66	Pre-operative Arrhythmia	1 = No
		2 = Yes
PRE_Inotropicos_pre_Adm1_67	Inotropic Use in the Pre-operative Period	1 = No
		2 = Yes
PRE_Vent_mec_Pre_Adm1_68	Pre-operative Mechanical Ventilation	1 = No
		2 = Yes
PRE_Traqueo_pre_Adm1_69	Pre-operative Tracheostomy	1 = No
		2 = Yes
PRE_Hipotiroidismo_pre_Adm1_70	Pre-operative Hypothyroidism	1 = No
		2 = Yes
PRE_Diabetes_Pre_Adm1_71	Pre-operative Diabetes	1 = No
		2 = Yes
PRE_Endocardite_pre_Adm1_72	Pre-operative Diagnosis of Endocarditis	1 = No
		2 = Yes
PRE_Sepsis_pre_Adm1_73	Sepsis in the Pre-operative	1 = No
		2 = Yes
PRE_Convulsao_pre_Adm1_74	Pre-operative Seizure	1 = No
		2 = Yes
PRE_Alt_Neuro_pre_adm1_75	Neurological Changes in the Pre-operative	1 = No
		2 = Yes
PRE_Disf_renal_pre_Adm1_76	Pre-operative Renal Dysfunction	1 = No
		2 = Yes
		3 = No information
PRE_Hipert_Pulm_pre_Adm1_77	Pre-operative Pulmonary Hypertension	1 = No
		2 = Yes
		3 = No information
PRE_Gastro_pre_Adm1_79	Pre-operative Gastrostomy	1 = No
		2 = Yes
		3 = No information
PRE_ECMO_VAD_pre_80	Pre-opeative ECMO Need	1 = No
		2 = Yes
		3 = No information
PRE_UTI_Previa_2sim_1nao_adm1_81	Previous ICU Admission	1 = não
		2 = sim
PRE_peso_kg_adm1_82	Patient Weight on the Surgery Date	1 = from 1.7 to 13.1 Kg
		2 = from 13.2 to 24.6 Kg
		3 = from 24.7 to 36.1 Kg
		4 = from 36.2 to 47.6 Kg
		5 = from 47.7 to 59.1 Kg
		6 = from 59.2 to 70.6 Kg
		7 = de 70.7 to 82.1 Kg
		8 = from 82.2 to 93.6 Kg
		9 = de 93.7 to 105.1 Kg
		10 = over 105.1 Kg
PRE_estat_cm_adm1_83	Patient Height on the Surgery Date	1 = up to 51 cm
		2 = from 52 to 66 cm
		3 = from 67 to 82 cm
		4 = from 83 to 97 cm
		5 = from 98 to 112 cm
		6 = from 113 to 128 cm
		7 = from 129 to 143 cm
		8 = from 144 to 159 cm
		9 = from 160 to 174 cm
		10 = above 175 cm
PRE_IMC_adm1_84	Body Mass Index on the Surgery Date	1 = from 6.3 to 9.6
		2 = from 9.7 to 13.0
		3 = from 13.1 to 16.4
		4 = from 16.5 to 19.8
		5 = from 1.9 to 23.2
		6—from 23.3 to 26.6
		7 = from 26.7 to 30.0
		8 = from 30.1 to 33.4
		9 = from 33.5 to 36.8
		10 = from 36.9 to 39.1
PRE_SC_adm1_85	Body Surface on the Surgery Date	1 = from 0.2 to 0.4 m^2^
		2 = from 0.5 to 0.7 m^2^
		3 = from 0.8 to 1.0 m^2^
		4 = from 1.1 to 1.3 m^2^
		5 = from 1.4 to 1.6 m^2^
		6 = from 1.7 to 1.9 m^2^
		7 = from 2.0 to 2.3 m^2^
		8 = over 2.3 m^2^
PRE_IDADE_CIRURGIA_dias_adm1_90	Age on the Surgery Date	1 = from 0 to 2,432 days
		2 = from 2,433 to 4,864 days
		3 = from 4,864 to 7,295 days
		4 = from 7,296 to 9,727 days
		5 = from 9,728 to 12,159 days
		6 = from 12,160 to 14,590 days
		7 = from 14,591 to 17,022 days
		8 = from 17,023 to 19,454 days
		9 = from 19,455 to 21,885 days
		10 = from 21,886 to 24,316 days
DESFECHO_FINAL_OBITO_89	Death	1 = Hospital discharge
		2 = Hospital death

### Outcome variables

The outcome variable of interest was hospital mortality, defined as death in the hospital or within 30 days of cardiac surgery, as defined by STS [10].

### Data analysis

The experiments were performed on an Intel® Core ™ i7-7700HQ 2.80GHz notebook, 16.0 GB of RAM, under the Windows 10 platform. Moreover, for the manipulation, analysis and training of the algorithms, Python 3.7.1 software and Numpy, Pandas, Matplotlib, Seaborn, Scikit-Learn, Imblearn and PyTorch libraries were used.

**Fig 1:**
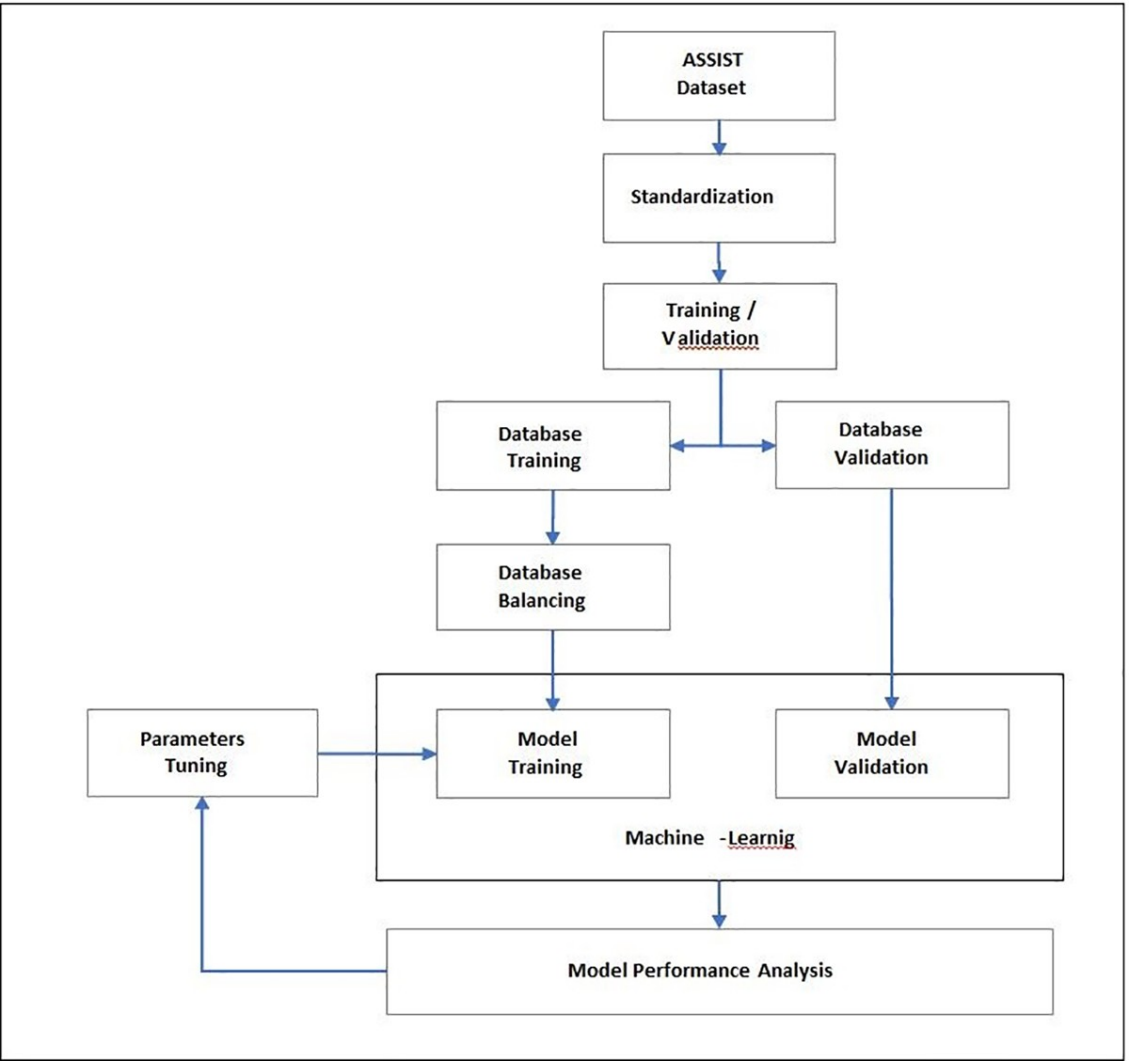
Steps performed in the model development.

The forecasting model development included the following steps: preparation of the InCor data set, normalization or standardization of the variables, division of the data into training and validation sub-sets, balancing of the training set, training and algorithm adjustments, and finally, measuring the model's forecast performance. Fig 1 presents this procedure sequence and subsequent texts define and further explain each step.

The Department of Cardiovascular Surgery-Pediatric Cardiac Unit, Heart Institute of University of São Paulo Medical School—InCor provided the data set used in this study and its technical expertise. The data set, extracted from the ASSIST database, contains the history of 2,240 cardiac surgeries performed on patients with heart disease from 2014 to 2018. This information was organized into 84 variables, many derived from the international RACHS and Aristotle risk scores checklists, including continuous, quantitative or categorical qualitative parameterized fields, as detailed in Table 2. We defined the objective variable as “Final Outcome” and tabulated it 0 or 1, with 0 for “Hospital Discharge” and 1 for “Hospital Death”.

**Table 2:** Summary of methods and techniques.

*Methods and Techniques*	*Bagged Decision Trees (BDT)*	*Random Forest* (RF)	*Stochastic Gradient Boosting (SGB)*	*Extra Trees (ET)*	*AdaBoost Classification* (ABC)	*Multilayer Perceptron (MLP)*
***ASSIST Database*** *(select variables)*	84 variables (all variables)	42 variables (Recursive Feature Elimination Method)	42 variables (Recursive Feature Elimination Method)	42 variables (Recursive Feature Elimination Method)	42 variables (Recursive Feature Elimination Method)	84 variables (all variables)
***Standardization*** *(method)*	Yes	Yes	Yes	Yes	Yes	Yes
***Separation in Training and Validation*** *(method)*	K-Fold Cross Validation	K-Fold Cross Validation	K-Fold Cross Validation	K-Fold Cross Validation	K-Fold Cross Validation	Stratified K-Fold Cross Validation
***Data Set Balancing*** *(method)*	Over-sampling by the algorithm	Over-sampling by the algorithm	Over-sampling by the algorithm	Over-sampling by the algorithm	Over-sampling by the algorithm	No
***Parameter Tuning*** *(technique)*	GridSearchCV and Randomized SearchCV	GridSearchCV and Randomized SearchCV	GridSearchCV and Randomized SearchCV	GridSearchCV and Randomized SearchCV	GridSearchCV and Randomized SearchCV	Experimental Method

As the first step to train of the algorithm, it was necessary to evaluate the need for normalization or standardization of the variables. Indeed, many machine-learning algorithms perform better or converge more quickly when the resources are on a relatively similar scale or close to the normal distribution, as for example, in Linear Regression, Logistic Regression, K-Nearest Neighbors Algorithm (KNN), Artificial Neural Networks (RNA), Support Vector Machines with radially polarized core (SVM), Principal Component Analysis (PCA) and Linear Discriminant Analysis (LDA) [18–21].

The data set partition for training and testing (validation) was separated by the forecasting statistic adjusted model. The training set was then used to train the model and the test set (validation) was used to evaluate the model's performance. However, this approach without adjustments could have led to problems of variance using the same algorithm, with scenarios where precision obtained in one test is different from the precision obtained in another test set. To minimize variance and ensure better performance of the machine learning models [22,23], Hold-out and K-Fold Cross Validation techniques were compared, regarding our model purpose and the size of the dataset. The Hold-out method divides the data set into two parts, training and testing, while the K-Fold Cross Validation method divides the data set into K parts of equal size, also called folds. The training process was then applied to all folds, with the exception of one fold that was used as a test set in the validation process, where, finally, the measure of performance was the average of all performance tests for all folds. The advantage of this K partition method is that the entire data set is trained and tested, reducing the variation of the chosen estimator. This guarantees a more accurate forecast and less bias from the positive rate estimator [23,24].

As in this study, the 10-fold K-Fold Cross Validation method (K = 10) has shown good performance in several health-related studies with low variability between the training and test sample [16,25–29].

Another source of results variance we analyzed was derived from unbalance within data categories included, such as, for example, some diagnostic variables (e.g. small numbers of rare conditions). Indeed, in data sets it is common to observe large differences in the percentage of representativeness in the classes studied. For instance, in the InCor’s study data set, we observed 10.8% patients dying after surgery versus 89.2% who survived. When the classification categories are not equally represented, it is said that the data set is unbalanced [30,31].

Conventional algorithms tend to be biased towards the majority class because their loss functions try to optimize quantities such as error rate, disregarding data distribution. In the worst case, minority examples are treated as outliers of the majority class and ignored, causing the model to be trained only to identify the majority class, which for this study it would lead to failure to classify a patient's risk of death.

The InCor’s data set is unbalanced, that is, there is a 1:9 ratio between mortality and post-surgery survival [30,31]. In some algorithms, if this effect is not addressed, there would be a false interpretation of the model's performance, which was not desirable since the study’s aim is to identify and understand the risk of death, the minority class of the studied data set.

To reduce the impacts due to the unbalance, technical methods to do under-sampling or over-sampling the data set were used. The under-sampling techniques consisted of reducing the sample of its most representative category to increase the sensitivity of a classifier towards its minority class, while the over-sampling technique simply increased the sample of the minority category using statistical techniques to replicate minority samples, duplicating them or generating new samples from the actual samples [30].

Moreover, in the process of building the machine-learning model, it was necessary to evaluate algorithm performance, the model’s errors and its hits capacity. In this study, the model aims to accurately predict the risk of death of patients with congenital heart disease before cardiac surgery, so it is a binary classification problem. In binary classification problems there are several evaluation metrics [32]; the most common performance metrics in machine learning are Accuracy, Precision, Specificity, Sensitivity or Recall, and the ROC (Receiver Operating Characteristics) curve AUC (Area Under the Curve), also written as AUROC (Area Under the Receiver Operating Characteristics).

**Fig 2:**
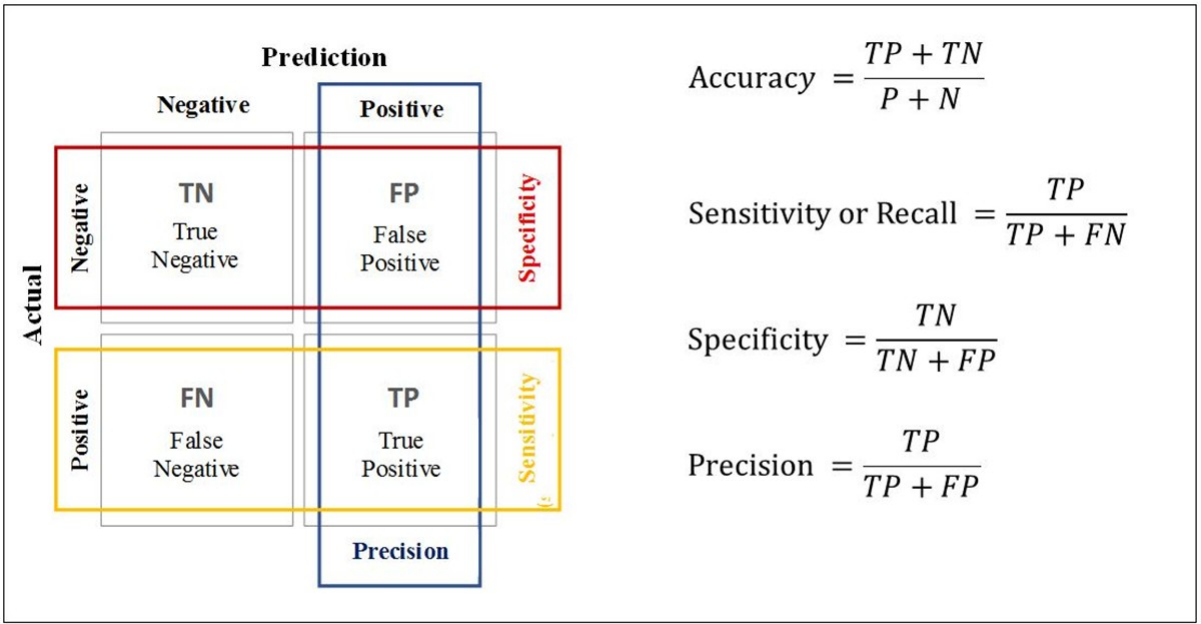
Confusion matrix.

Accuracy, Precision, Specificity, Sensitivity or Recall measurements were calculated using the Confusion Matrix (Fig 2).

The methods and techniques used in this study are summarized in Table 2.

## Ethical approval

This study is part of the larger ASSIST Registry project ("Estudo do Impacto dos Fatores de Risco na Morbimortalidade das Cardiopatias Congênitas: comparação entre estratos de risco quando duas escalas internacionais são aplicadas no Sistema Único de Saúde do Estado de São Paulo"), protocol CAAE: 31994814.0.3001.5440 approved by the Ethics Committee of the Heart Institute of University of São Paulo Medical School, São Paulo, Brazil. Because this study used solely the pre-established database by the larger ASSIST Registry project, the use of the patient’s informed consent forms was waived.

## Companion web site

A companion site was designed to contain additional, up-to-date information on the data set, model as well as a Web Application that can perform mortality predictions based on individual patient characteristics.

## Results

The predictive performance metrics of the machine learning algorithms tested in this study are shown in Table 3.

**Table 3:** Performance metrics of algorithms.

Algorithms	Accuracy	Precision	Recall	Specificity	ROC AUC	Average Precision
Bagged Decision Trees	86.2%	69.2%	70.6%	90.8%	92.6%	0.81
Random Forest	80.8%	54.7%	92.2%	77.5%	90.2%	0.73
Extra Trees	72.8%	44.7%	82.4%	69.9%	86.0%	0.69
AdaBoost	84.4%	65.4%	66.7%	89.6%	85.7%	0.71
Gradient Boosting	82.6%	59.7%	72.5%	85.5%	88.5%	0.70
NN MLP	90.2%	62.5%	20.8%	98.5%	84.6%	0.44

Table 3 shows that the Multilayer Perceptron (MLP) neural network obtained the highest levels of accuracy (accuracy) and specificity (specificity) in relation to the other studied algorithms, respectively 90.2% and 98.5%. On the other hand, it obtained the lowest sensitivity index (20.8%), ROC AUC (84.6%) and AP ((Average Precision) 0.44). These results demonstrate that the Multilayer Perceptron neural network achieved the best performance in the accuracy of survival forecasts, a fact reinforced with the specificity index (Specificity) of 98.5%. In contrast, its ability to identify patients at risk of death is the lowest among the models studied; only 20.8% of the total patients who died in surgery were identified as risk of death by the neural network.

Given our InCor’s unbalanced dataset [33,34], we used the ROC AUC and AP to analyze the performance of the models. The Bagged Decision Trees (BGT), Random Forest (RF) and Stochastic Gradient Boosting (SGB) algorithms stand out with the highest ROC AUC rates among the studied algorithms, respectively 92.6%, 90.2% and 88.5%. They also have the highest AP rates, 0.81 for Bagged Decision Trees (BGT), 0.73 for Random Forest (RF) and 0.70 for Stochastic Gradient Boosting (SGB). The sensitivity index (Recall) is another metric considered useful to subsidize decision making, where the 92.2% index for Random Forest (RF) is observed, the highest index among the studied algorithms.

In line with the objective of predicting the risk of pre-surgical mortality, the Bagged Decision Trees (BGT) and Random Forest (RF) algorithms stand out in the performance requirement. The Bagged Decision Trees (BGT) algorithm demonstrated better performance for predicting survival, specificity (specificity) of 90.8%, without giving up the ability to identify risk of death, sensitivity index (Recall) of 70.6%. While Random Forest (RF) stood out in its ability to identify risk of death, reaching a sensitivity index (Recall) of 92.2%.

**Fig 3:**
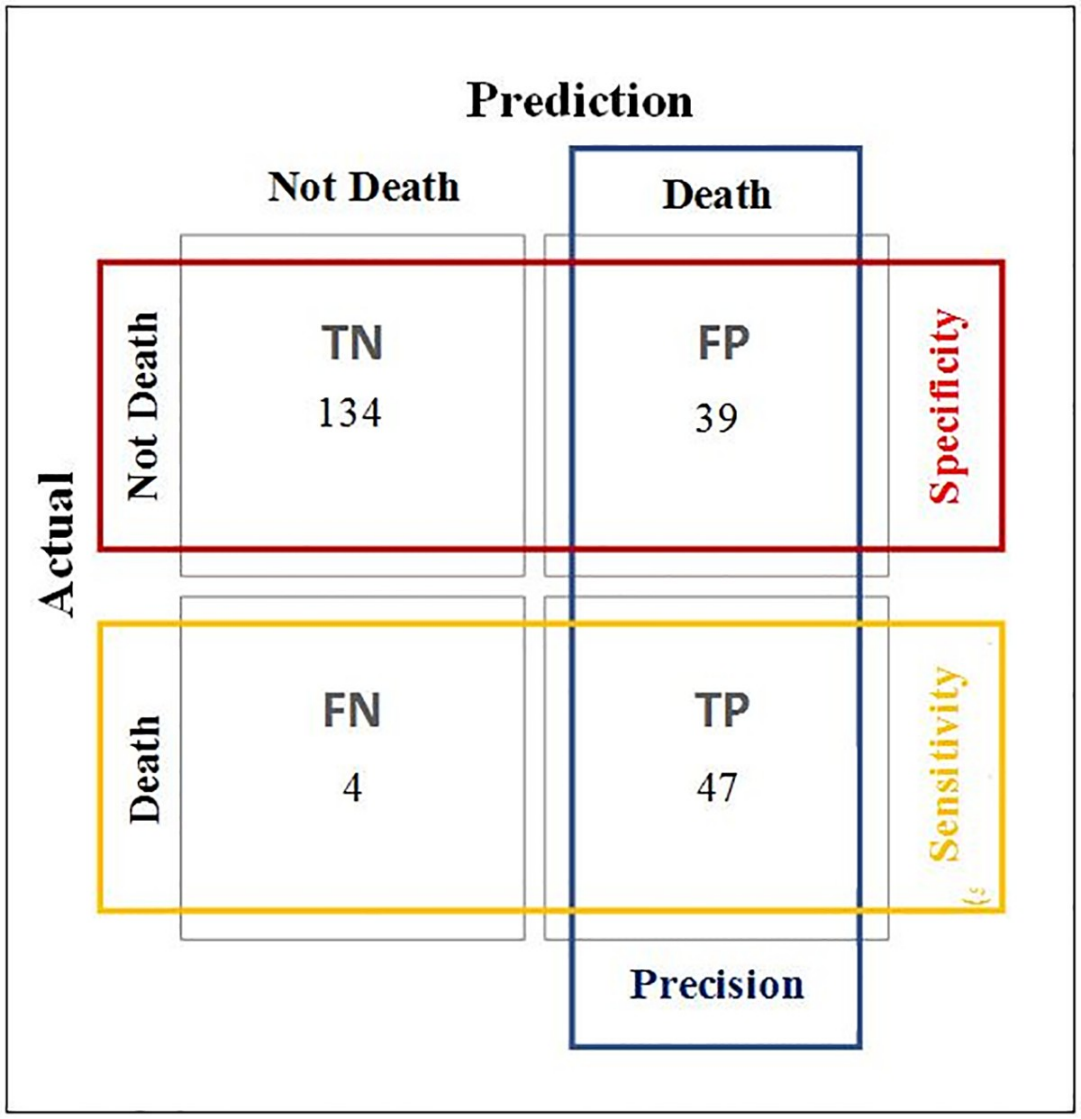
Confusion matrix of the RF algorithm.

**Fig 4:**
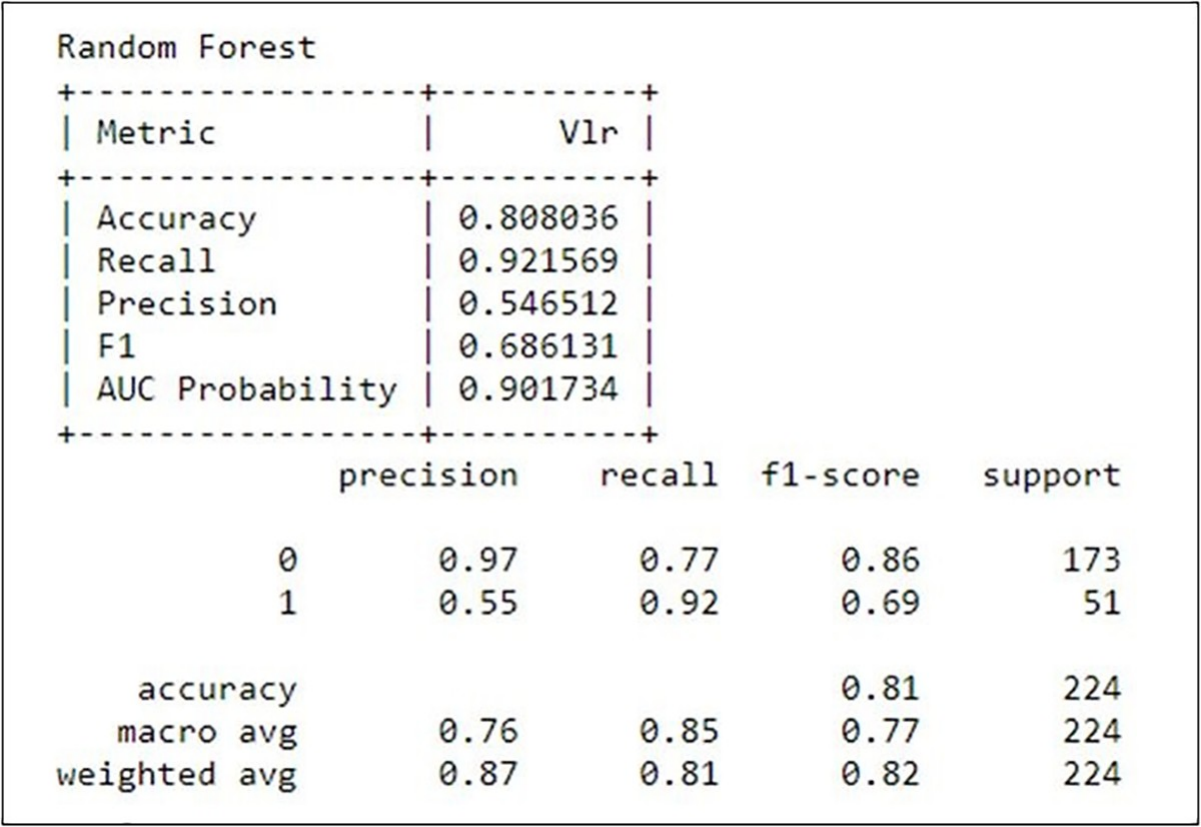
Performance metrics of the RF algorithm.

The data in the Confusion Matrix of the RF algorithm are from the test set (validation), in which it is possible to verify the number of observations with correct and predicted errors. It can be seen that 8 of the 51 patients who died were not predicted by the model (Fig 3). This matrix contains the information that was used to generate the model's Accuracy, Sensitivity or Recall, Specificity and Precision Indices (Fig 4).

In Fig 4, it can be seen that the RF model obtained an accuracy of 80.8%, sensitivity of 92.1% and precision of 54.6%.

**Fig 5:**
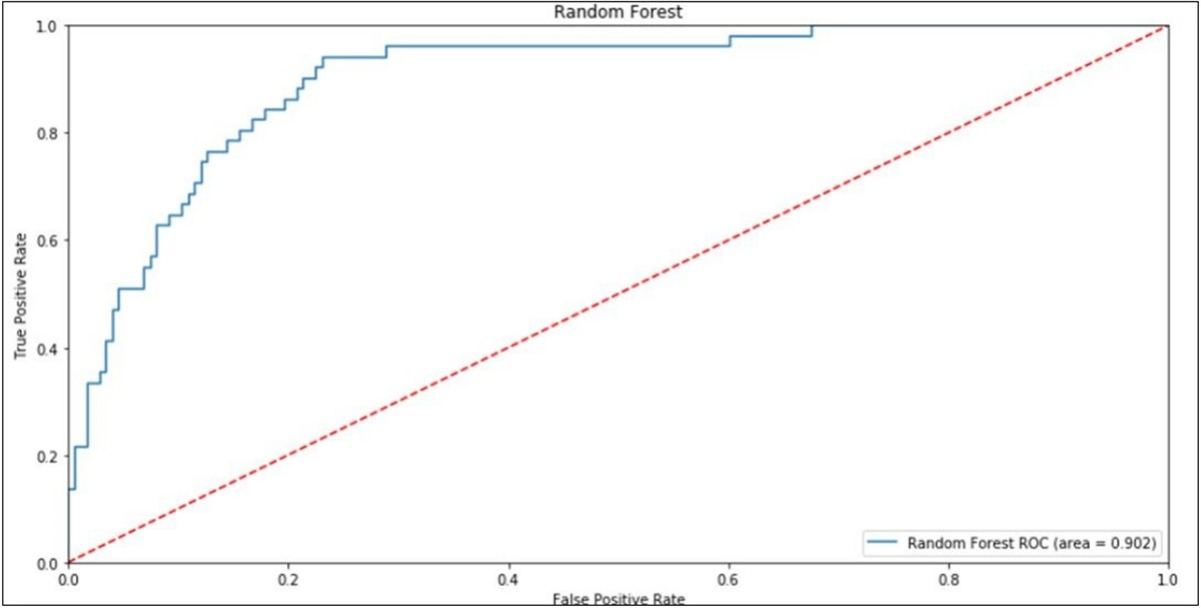
ROC AUC curve of the RF algorithm.

The ROC AUC (AUROC) curve is another model performance metric frequently used to support medical decision-making [35] increasingly adopted in the machine learning research community [36]. The ROC AUC curve of the RF algorithm is shown in Fig 5.

Fig 5 shows the rate of false positives on the horizontal axis and the rate of true positives on the vertical axis, and the plotted curve is the ROC curve. The area under the curve is called AUC (Area Under Curve) and indicates the model's ability to hit. The closer this index gets to 1, the greater the model's ability to hit its predictions. The Random Forest (RF) reached a rate of 90.2% of ROC AUC, information used to compare the performance of the other models and to support the decision of the cutoff values for the desired objective.

**Fig 6:**
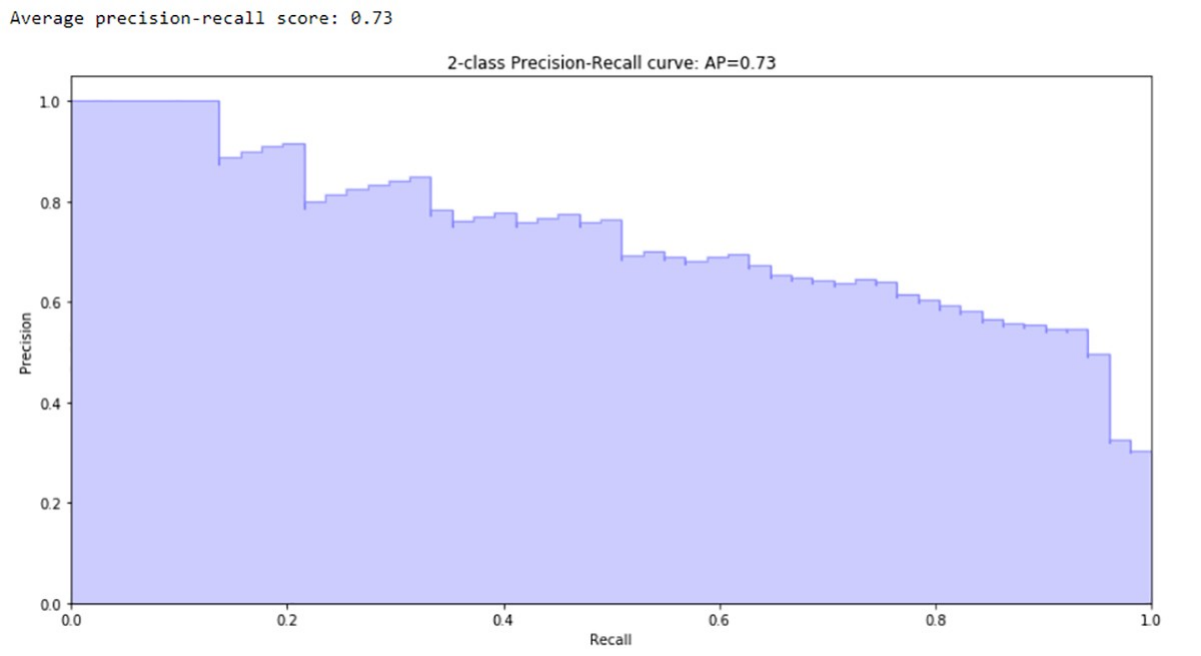
Precision-Recall curve of the RF algorithm.

Due to the InCor’s unbalanced dataset [33,34] and the warn of caution in the use of the AUROC it is recommended to include Precision-Recall Curves in decision-making (Fig 6).

The Precision-Recall Curve shows the correlation of those two indices. It is observed that the higher the Recall, the lower the Precision of the generated model. It is important information for the cutoff point of the model. The highest precision is obtained in detriment of the sensitivity, or the opposite. The AP index is also calculated, which in the RF model was 0.73.

The variables importance and influence analysis using the Random Forest (RF), Stochastic Gradient Boosting (SGB), Extra Trees (ET) and AdaBoost Classification (ABC) algorithms is presented, with the resulting magnitude each variable affected the risk of death, in Table 4.

**Table 4:** Importance of predictor variables in the outcome.

	Significance			
Variable	Random Forest (RF)	Stochastic Gradient Boosting (SGB)	Extra Trees (ET)	AdaBoost Classification (ABC)
Previous ICU Admission	22.2%	11.4%	8.9%	5.3%
Diagnostic Group	15.8%	2.2%	2.4%	5.3%
Patient Height at Surgery	11.6%	17.7%	5.2%	9.3%
Arterial Oxygen Saturation	5.5%	10.0%	5.7%	5.3%
Left Heart Hypoplasia Syndrome	6.5%	13.6%	2.6%	1.3%
Body Mass Index in Surgery	6.3%	2.7%	3.5%	5.3%
Preoperative Hematocrit	0.9%	4.0%	4.1%	2.7%
Pulmonary Atresia	4.3%	3.1%	2.8%	1.3%
Patient Weight in Surgery	2.4%	0.0%	1.6%	6.7%
Number of Previous Procedures	0.2%	1.5%	2.0%	6.7%
Diagnostic Category	1.6%	3.1%	2.6%	2.7%
Number of Hospitalizations	0.9%	0.7%	2.6%	5.3%
Body Surface in Surgery	5.4%	0.3%	3.3%	0.0%
Age at Surgery Date	1.9%	1.6%	1.1%	2.7%
Diagnosis of Congenital Heart Disease in Prenata Care	1.4%	0.5%	2.5%	2.7%
Preoperative Diagnosis of Endocarditis	0.9%	1.5%	1.1%	1.3%
Preoperative Mechanical Ventilation	0.3%	1.4%	1.8%	1.3%
Sex	0.5%	0.0%	2.9%	1.3%
Tetralogy of Fallot	0.7%	0.0%	1.0%	2.7%
Single Ventricle	1.3%	2.1%	0.9%	0.0%
Preoperative Hypothyroidism	0.3%	0.4%	0.8%	2.7%
Preoperative ECMO Need	0.1%	1.9%	0.8%	1.3%
Prematurity	0.3%	1.2%	2.6%	0.0%
Provider	0.5%	0.0%	2.2%	1.3%
Cardiomyopathy	1.3%	0.8%	1.6%	0.0%
Other Syndromes	0.3%	1.1%	0.7%	1.3%
Son of Diabetic Mother	0.4%	0.3%	2.6%	0.0%
Undergoing Previous Procedures	0.4%	0.3%	1.1%	1.3%
Down's Syndrome	0.1%	0.5%	1.0%	1.3%
Arterial Communication	0.3%	0.0%	2.2%	0.0%
Interventricular Communication	0.3%	0.0%	2.1%	0.0%
Non-Cardiac Abnormality—Chromosomal Syndrome	0.5%	0.3%	1.4%	0.0%
Preoperative Seizure	0.9%	0.0%	0.0%	1.3%
Dual Right Ventricular Outflow Tract	0.5%	0.0%	0.0%	1.3%
Previous Surgery	0.6%	0.0%	1.2%	0.0%
Persistence of the Arterial Canal	0.2%	0.0%	0.0%	1.3%
Use of Inotropic Preoperatively	0.4%	0.5%	0.0%	0.0%
Non-Cardiac Abnormality—Malformations	0.4%	0.0%	0.0%	0.0%
Total Anomalous Drainage of the Pulmonary Veins	0.3%	0.0%	0.0%	0.0%
Aortic Valve Disease	0.2%	0.0%	0.0%	0.0%
Preoperative Renal Disfunction	0.1%	0.0%	0.0%	0.0%

Table 4 shows that some variables are listed as the most important in the four outstanding studied algorithms, such as: Previous ICU admission, Diagnostic Group, Patient Height at the Time of Surgery, Arterial Oxygen Saturation, Hypoplasia of the Left Heart and the Body Mass Index in Surgery. These variables together represent 67.8% of the importance of the risk of death in the Random Forest (RF) model, 57.6% in the Stochastic Gradient Boosting (SGB), 28.4% in Extra Trees (ET) and 32.0% in AdaBoost Classification (ABC).

## Discussion

In recent research on individualized mortality prediction models, it is observed that the use of machine learning techniques can be a tool to support medical decision making. Research in this field has been increasing in recent years [16]. These techniques have shown better performance compared to traditional techniques, such as logistic regression, [15,25,37], including in mortality prediction studies [15].

The experiment was started following the steps described in Fig 2. With the normalized data set, the training and test samples (validation) were separated, using the K-Fold Cross Validation method 10 times (K = 10), where 90% of the sample was separated to train the machine learning algorithms and the rest separated for the validation step. Then, the need to balance the data set was assessed, since there is an unbalanced set, a 1:9 ratio between mortality and post-surgery survival and the search for greater predictability of risk of death. When balancing was necessary, different methods of under-sampling or over-sampling were tested to adjust the distribution of the training sample categories.

With the training and test samples (validation) separated and adjusted, the training step of the algorithm began. In the process of training the algorithms, several parameter configurations were tested, always seeking to minimize the generalization errors, either by overfitting or under fitting [38].

In order to optimize the generalization of the algorithm, the ROC AUC (AUROC) and Average Precision indexes were maximized, aiming to obtain the best forecasting assertiveness and minimize the error of not signaling a patient at high risk of mortality.

Among the six machine learning algorithms studied, it was observed that the Bagged Decision Trees (BGT) and Random Forest (RF) algorithms stood out in predicting mortality in relation to the other studied algorithms. In this selection to decide which one of the algorithms best fulfilled the mortality prognosis, we considered the implementation complexity and the possibility to understand the impact of the variables on the risk of death. Based on these parameters, the Random Forest (RF) algorithm outperformed and made possible to analyze the importance of variables, a function not present in Bagged Decision Trees (BDT). It was also observed that the Random Forest (RF) algorithm did not use all the variables of the data set in the generation of the model, thus, it reduced the implementation complexity. Moreover, the Random Forest (RF) can be highlighted for its precision, ease of training, and adjustments [21].

To the best of our knowledge, this is the first individual’s CHD mortality prognosis ascertainment using AI.

Our AI derived outcomes analyses are in line with the aggregated international scientific literature published. The variables listed as the most important, where the representativeness of “hospital death” is greater with more severe CHD diagnosis, indeed, agrees with the STS core aggregated data published. However, the STS aggregated data more recently published [10] has excluded low weight or out of -7.0 to 5.0 Z score range neonates present in InCor’s patient population due to malnutrition, problems not prevalent in developed countries.

## Conclusions

This study suggests the use of Random Forest (RF) as a model of individual death prediction for cardiac surgery in patients with congenital heart disease. The prediction results of Random Forest (RF) corroborate that machine learning algorithms can assist clinical specialists, patients and family members to analyze the risks associated with a possible cardiac surgical intervention.

Understanding which diagnoses and variables impact the probability of mortality of a patient with congenital heart disease, when proposed to be submitted to a cardiac surgical intervention, allows clinical specialists to understand the risks associated with a surgical intervention, provide information to support the decision of health professionals and family members of patients.

Analyzing the variables listed as the most important, it was observed that the representativeness of “hospital death” is greater in patients up to 66 cm in height and BMI below 13.0. In addition, the “hospital death” probability declines with the increase in the arterial oxygen saturation index, allowing focusing the action and medical intervention to mitigate the risk of death.

In the patients’ cluster with previous ICU stay or with prior hospitalization before surgery, it was observed the highest proportion of deaths than the patients who did not needed such admissions. In-depth analysis of the effects of this variable is timely in understanding the risks of death and may be the target of studies in future research.

Accordingly, the most severe diagnosis groups have a higher percentage of death than others, for example the left heart hypoplasia syndrome.

It is, thus, opportune to direct specific studies for these groups and variables that can direct actions to mitigate the risk of death.

As a model-based mortality prediction tool, the CngtSCORE model can be accessed through Web browsers and smartphones.

## Perspectives

Future research can evaluate new machine-learning algorithms or even test new variables and diagnoses, as well as an in-depth analysis of the effects of these variables in understanding the risks of death in patients with congenital heart disease undergoing cardiac surgery.

In addition, given the transition of pediatric care into adult life, the continuous evolution of treatment strategies, and the relatively long life expectancies of survivors of cardiac interventions, new machine learning algorithms may compare the long-term efficacy of different treatment strategies.

## Supporting information

S1 Data (XLSX)
